# Therapeutic Effects of Medicinal Mushrooms on Gastric, Breast, and Colorectal Cancer: A Scoping Review

**DOI:** 10.7759/cureus.37574

**Published:** 2023-04-14

**Authors:** Amit Dan, Robyn Swain, Seigna Belonce, Robin J Jacobs

**Affiliations:** 1 Department of Nutrition, Nova Southeastern University Dr. Kiran C. Patel College of Osteopathic Medicine, Fort Lauderdale, USA; 2 Departments of Research/Health Informatics/Nutrition, Nova Southeastern University Dr. Kiran C. Patel College of Osteopathic Medicine, Fort Lauderdale, USA

**Keywords:** agaricus sylvaticus, breast cancer, colorectal cancer, coriolus versicolor, gastric cancer, lentinus edodes, medicinal mushroom, polysaccharide-k, psk, turkey tail

## Abstract

Cancer is the leading cause of mortality globally. With anticancer medications causing severe adverse effects, understanding the role of alternative and efficacious anticancer treatments with minimal or no side effects can be beneficial. Edible mushrooms have been associated with certain health benefits and exhibit a broad range of pharmacological activities, including anti-inflammatory and immunomodulating activities. The anticancer potential of different mushrooms is now being tested. The goal of this scoping review was to discuss the most recent and available evidence on the therapeutic uses of medicinal mushrooms in cancer treatment, specifically those cancers with some of the highest mortality rates (i.e., gastric, breast, and colorectal cancer). Randomly controlled trials, clinical trials, and retrospective cohort studies (with placebo group) with human subjects published between 2012-2023 were searched using the databases Embase, Ovid MEDLINE, Cumulative Index to Nursing and Allied Health Literature (CINHAL), and Alt HealthWatch. The initial search yielded 2,202 articles. After removing 853 duplicate citations, 1,349 articles remained and were screened for study eligibility and accessibility, leaving 26 articles. The inclusion and exclusion criteria were then used to assess the remaining 26 full-text articles and nine articles were selected for the final review. The characteristics of the nine studies reported the efficacy of medicinal mushrooms *Lentinus edodes* (Shiitake), *Coriolus versicolor* (Turkey Tail), and *Agaricus Sylvaticus *(Scaly Wood), in treating symptoms, medication side effects, anti-tumor effects, and survival outcomes in gastric, breast, and colorectal cancers. Findings from this review suggest that medicinal mushrooms have the potential to prevent lymph node metastasis, prolong overall survival, decrease chemotherapy-induced side effects (e.g., diarrhea, vomiting), affect the immune system, and help maintain immune function and quality of life in patients with certain cancers. More research is needed with human subjects using RCTs with larger samples to ensure accurate outcomes and ascertain the most efficacious dosages.

## Introduction and background

Medicinal mushrooms have been used for centuries in traditional medicine for their numerous health benefits. Recently, studies have shown that certain types of mushrooms have potent anticancer properties, making them a potential complementary therapy for cancer patients. Edible mushrooms have long been associated with certain health benefits, not excluding decreasing the risk of cancer. The numerous mushroom species are still very much understudied, but many of their novel bioactive compounds are currently being isolated to further the research. As of late, the benefits of mushrooms are being systematically studied. They contain a class of polysaccharides such as beta-glucans and other products that are converted into sustainable pharmaceutical compounds [1]. Although evidence exists to support their therapeutic effects, research is still needed to translate mushroom-based substances into patient care. There is research to support medicinal mushrooms’ impact on immune health [2]. Several types of mushrooms have been studied to ascertain their therapeutic benefits for viral and bacterial infections, as well as cancer [3]. 

There are published reports regarding the consumption of mushrooms and mushroom-based extracts that have indicated an association between mushroom intake and lowered cancer incidence and increased survival rates [4-7]. While there is limited data regarding lung cancer risk factors and mushroom consumption, it is crucial to discuss the studies that show promise in lung cancer risk reduction, prevention, and symptom relief for patients undergoing allopathic treatments such as chemotherapy and radiation. For example, molecules such as lentinans, found in the shiitake mushroom, have been linked to improved efficacy in lung cancer treatment and decreased nephrotoxicity in patients exposed to nephrotoxic lung cancer drugs [8]. Considering the possible adverse effects of chemotherapeutics combined with growing awareness of potentially beneficial alternative remedies to prevent or mitigate the effects related to cancer and its treatments, further investigation of the potential benefits of medicinal mushrooms on lung cancer is warranted.

Gastric cancer, commonly known as stomach cancer, is ranked the fifth most common neoplasm and the third most deadly cancer globally and has high morbidity and a 5-year survival rate of less than 30% [9,10]. Although the gastric cancer mortality rate has decreased in the last few years, gastric cancer remains an ongoing global health crisis [11]. Although the etiology of gastric cancer has not been confirmed, one becomes susceptible to the disease with exposure to *Helicobacter pylori* (*H. pylor*i) infection, radiation, dietary and behavioral habits, obesity, gastroesophageal reflux disease, low socioeconomic status, and genetic predisposition [12]. The standard of care for gastric cancer patients remains surgery and perioperative and adjuvant chemotherapy and chemoradiation; it has been indicated to improve treatment outcomes [13]. Polysaccharides from* P. igniarius *have been seen to diminish tumors and contain antiproliferative, antimetastatic, and antimutagenic effects in vitro [14,15]. Moreover,* P. igniarius* consists of a hispolon which has been found to have significant effects against various types of cancer, including gastric cancer [15]. Other mushroom species, such as *Coriolus versicolor* (Turkey Tail), have been under study as possible adjuvant therapy to strengthen the immune system when given with standard cancer [2,16-18].

There is research suggesting that some mushrooms such as *Fusharium oxysporum *and *Agaricus visporus*, have chemoprotective agents that help the body combat breast cancer [2]. *Lentinus crinitus* (also known as Fringed Sawgill mushroom), was found to be a nuclear factor-κB (NF-κB), a transcription factor that regulates the expression of genes associated with cell survival, proliferation, and differentiation. Fringed Sawgill affects inflammatory and immune responses and has been known to help control breast cancer growth [8]. Collating and assessing published reports on the effects of certain mushrooms can give us an insight into how these may be used to treat breast cancer.

Colorectal cancer (also known as colon or rectal cancer) is an illness in which the cells of the rectum or colon grow out of control and spread to other body parts [19]. There are many risk factors that could increase the chance of one developing colorectal cancer such as older age, being Black, family history, having a low-fiber diet, and having a sedentary lifestyle [20]. Medical solutions include surgery, radiation therapy, and drug treatment to control and or eradicate cancer [20]. While still in the exploration phases, *agaricus sylvaticus* is a medicinal mushroom that has been shown to have anticancer properties, improve immunological parameters, and reduce glycemia levels [21]. Moreover, medical mushrooms have not been reported to have harmful effects in the treatment of colorectal cancer compared to synthetic chemotherapies but may produce significant beneficial effects [22].

There is evidence to suggest that, in general, medicinal mushrooms are safe for human consumption. While their detrimental effects have been largely unexamined, there is evidence to suggest that some species may pose some health risks (e.g., allergic reactions, hypoglycemia in some people with diabetes, liver failure) and side effects (e.g., itching, dizziness, nausea, stomach upset, rash, and dry mouth) [23-25], not unlike many pharmacologic medications. Toxicity can also arise when medicinal mushrooms are contaminated with heavy metals, pesticides, or other harmful substances. Many medicinal mushrooms are grown in China, where there have been reports of contamination with heavy metals such as lead and cadmium, as well as pesticide residues, which can pose serious health risks [26]. However, due to their antimicrobial, anti-tumor products and anti-cancer benefits, medicinal mushroom treatment may be among the potential candidates for a more standard treatment modality if explored as a chemo-preventative pharmaceutical product. More scientific investigation is needed to help understand the complexity of the balance between the advantageous and potentially harmful effects of medicinal mushroom intake.

## Review

Methods

The goal of this scoping review was to collate the current published research on medicinal mushrooms used for cancer, specifically those cancers with high mortality rates by conducting a scoping review of the available recent evidence.

Design

A scoping review was conducted, incorporating summaries, explanations, and interpretations from randomized controlled trials (RCTs), clinical trials (CTs), and retrospective cohort studies (with placebo group) to address the review question, “What are the therapeutic effects of medicinal mushrooms with regard to lung, gastric, breast, and colorectal cancers?” This method allowed a review to extract various data and summarize them in a way that is transparent, systematic, and useful. The review was performed as suggested by Arksey and O’Malley which proposes five stages of conduct: 1) specify the research question, 2) identify relevant literature, 3) select studies, 4) map out the data, and 5) summarize, synthesize, and report the results [27].

Search Strategy and Identification of Studies

An online search utilizing Embase, Ovid MEDLINE, Cumulative Index to Nursing and Allied Health Literature (CINHAL), and Alt HealthWatch databases was performed to collate the published research on medicinal mushrooms and their potential uses for lung, breast, colorectal, and gastric cancer. The inclusion/exclusion criteria were established before embarking on the review. Articles were selected for inclusion if they were in English, published between January 2012 - January 2023, randomized controlled trials, controlled studies, or retrospective cohort studies (with a control group), and included humans only. Studies that did not include experiments or retrospective cohort studies reports were excluded (e.g., book chapters, editorials, conferences, abstracts, and erratum). The combined keywords “medicinal mushroom” OR “edible mushroom” OR “garicales” OR “mushroom” AND “lung cancer” OR “pulmonary cancer” AND stomach cancer” OR “gastric cancer” AND “colorectal cancer” AND “breast cancer” were used for article searching. Using Boolean operators, the search terms were used in all four databases. The detailed search queries for each of the four databases are in the Appendix.

Search Outcome

**Figure 1:**
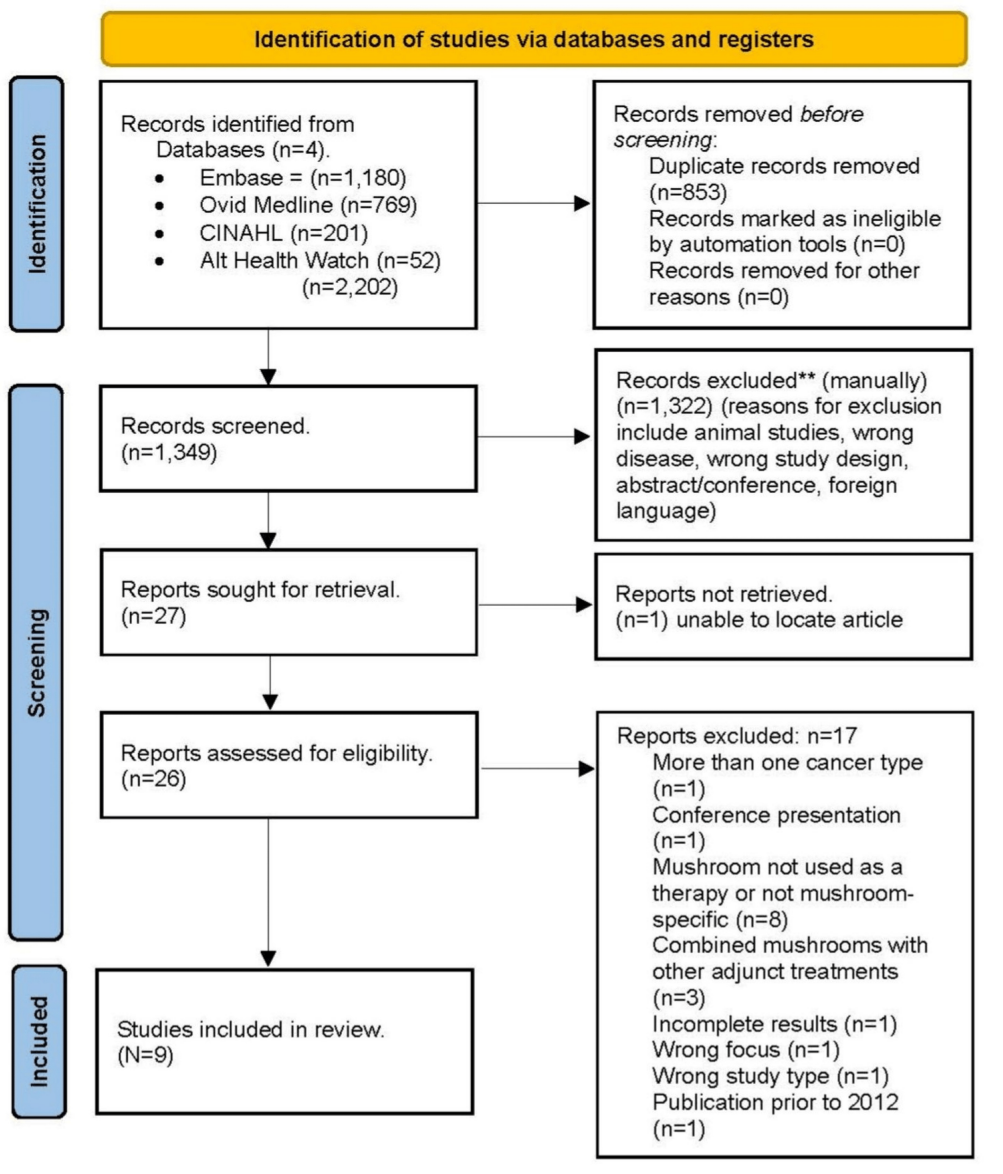
PRISMA flow diagram. PRISMA: Preferred Reporting Items for Systematic Reviews and Meta-Analyses.

Using the search criteria, the initial search yielded 2,202 articles. After deleting 853 duplicate citations, 1,349 articles remained and were screened for study eligibility. Records that were book reviews, editorials, errata, or conference abstracts were excluded (n=1,322), leaving 27 articles for the next level of review. After assessing the 27 citations for full-text accessibility, one article was removed (unable to locate it), leaving 26 articles for further screening. The inclusion and exclusion criteria were then used to assess the remaining 26 full-text articles (five of which pertained to lung cancer). All five articles regarding lung cancer did not meet the study’s inclusion criteria, and thus no evidence on the therapeutic effects of mushrooms for lung cancer was retained for the final review. Nine articles were selected for further critical appraisal (Figure 1).

Quality Appraisal

The Joanna Briggs Institute Critical Appraisal Tools were used to evaluate the possibility of bias based on analysis of the methodology used in the included studies for this scoping review. The Joanna Briggs (JB) checklists allowed for the classification of the nine articles based on the risk of bias. Articles passing < 50% of the checklist criteria were considered as high risk, articles passing 50-75% were intermediate risk, and articles passing > 75% were considered low risk for bias. The articles were analyzed by using the JBI checklist that corresponded most closely with the study methodology. All nine articles were considered low risk of bias (Figure 1).

Data Extraction

For all nine articles retained for review, the citation details (author, title, journal, year of publication) were entered into an Excel spreadsheet to document the scientific method, the goal of the study, cancer type, mushroom type, description of the sample, reported findings, and study limitations. Articles were then grouped by cancer type and sub-grouped by mushroom type. 

Results 

Table 1 reports the characteristics of the nine studies in the review. 

**Table 1:** Summary table of the nine articles included in the review PSK: polysaccharide-K; UFT/LV: oral adjuvant uracil and tegafur plus leucovorin; DFS: disease-free survival

Study	Purpose	Sample	Methods / Study Design	Type of Mushroom	Major Findings
GASTRIC CANCER					
Hsu et al. 2016 (28)	Elucidate long-term outcomes obtained in gastric cancer patients treated by adjuvant immunochemotherapy and how this is influenced by PD-L1 (a protein that helps keep the body’s immune responses under control) expression based on a large-scale surgical series.	918 patients with stages II and III gastric cancer undergoing curative gastrectomy and receiving adjuvant chemotherapy.	Retrospective review of patient charts classified into four cohorts stratified by PD-L1 expression and PSK administration, namely PD-L1, PSK. In addition, another independent cohort of 20 patients undergoing radical gastrectomy was prospectively recruited to check their immunological cells of sera before and 2 months after PSK administration. Meta-analysis performed.	PSK (from Trametes versicolor, also known as Coriolus versicolor and Turkey Tail mushroom).	The results collectively suggested that PSK serves as an immune modulator to enhance effectiveness of adjuvant chemotherapy in gastric cancer patients, providing survival benefits for patients with stage IIIA/IIIB disease, especially in PDL negative group.
Ito et al. 2012 (29)	Evaluate the expression of major histocompatibility complex (MHC) class I in gastric cancer patients who received PSK postoperative adjuvant immunochemotherapy and investigate the correlation between MHC class I expression and clinical outcomes.	The trial consisted of 349 stage II/III gastric cancer patients receiving adjuvant therapy after curative resection.	Controlled trial with patients in 2 treatment groups. The first group was given oral chemotherapy only; the second group was given chemotherapy in addition to PSK. Out of 349 patients, 225 patients with adjuvant chemotherapy were given only an oral fluoropyrimidine formulation; the other 124 patients were given oral fluoropyrimidine plus PSK as immunotherapy .	PSK (from Trametes versicolor, also known as Coriolus versicolor and Turkey Tail mushroom).	Survival‑prolonging effects from PSK when included in gastric cancer therapy in patients who have had curative resection, at high risk of recurrence (presence of lymph node metastasis and the absence of MHC class I expression). It was thought that PSK effects residual cancer cells that have eluded antitumor immune functions; potential to prevent lymph node metastasis.
Tanaka et al. 2012 (30)	Evaluate the effect of Protein-bound PSK on the overall survival of patients with gastric cancer.	254 patients with gastric carcinoma. Inclusion was limited to patients to whom curative surgery with postoperative adjuvant treatment with clinical stage II/III was offered.	Retrospective study of patients with gastric carcino split into a study group of PSK plus Fluoropyrimidine and control group of just Flouropurimidine therapy for approximately 16 months.	PSK (from Trametes versicolor, also known as Coriolus versicolor and Turkey Tail mushroom).	In the analysis of all patients, no significant differences were found in relapse-free or overall survival. Although, survival was improved in patients with multiple lymph node metastases following administration of PSK.
Wang et al. 2022 (31)	Evaluate the efficacy of PSK as an adjuvant therapy alongside chemotherapy in patients outside of Japan with gastric cancers who have already had a gastrectomy.	10,617 newly diagnosed gastric cancer patients who had a gastrectomy and were receiving adjuvant chemotherapy.	Retrospective historical study cohort with a control group.	PSK (from Trametes versicolor, also known as Coriolus versicolor and Turkey Tail mushroom).	The PSK group had a significantly longer survival than the control group. After adjusting for age, sex, urbanization, income, comorbidities and chemotherapy regimen, the use of PSK was still significantly associated with a reduction in the risk of death.
Qi et al. 2020 (10)	Determine the antitumor role of Huaier granules and the potential antitumor mechanism of Huaier polysaccharides in gastric cancer.	136 Stage IIb gastric patients were enrolled, with 54 in the Huaier+Tegafur Gimeracil Oteracil Potassium group, and 72 in the Tegafur Gimeracil Oteracil Potassium only group.	Retrospective cohort design. Dosage for patients that got oral Tegafur Gimeracil Oteracil Potas-sium was 60 mg. twice daily (capsule form) for the first 14 days, 3 weeks of a cycle within 1 year, with or without Huaier granule 20 mg. 3 times per day for 1 year.	Trametes robiniophila Murr (also known as Huaier, a sandy beige mushroom that grows on hard wood trees.	Patients in the study group showed to have greater DFS compared to the control group. Overall, Huaier polysaccharides promote anti-tumor and gastric cell apoptosis effectively.
BREAST CANCER					
Hangai et al. 2013 (32)	Explore the beneficial effects of active hexose-correlated compound (AHCC) on adverse events in patients receiving adjuvant chemotherapy for breast cancer.	Study consisted of 41 female patients treated with anthracyclines and taxanes for breast cancer (aged 31-61 years)	This was a retrospective cohort study (with control group) that surveyed medical records of women with breast cancer on adjuvant chemotherapy for which the treatment group received AHCC therapy; Patients who had taken AHCC during chemotherapy (1.0 g) by mouth post each meal. The placebo group did not receive AHCC.	Active hexose-correlated compound (AHCC), an extract of Lentinula edodes (Shiitake), which contains a polysaccharide called lentinan.	The AHCC group had significantly fewer neutrophil related events and less use of granulocyte colony-stimulating factor. The treatment group also experienced a higher (non-significant) rate of adverse events associated with c-glutamyl transpeptidase. AHCC may be able to lower the severity of neutropenia stemming from chemotherapy (including granulocyte-colony stimulating factor use during chemotherapy; AHCC also showed promise in lowering the toxicity of chemotherapy, allowing for increase in dosage breast cancer patients receiving chemotherapy.
Nagashima et al. 2017 (34)	Evaluate the effectiveness of Lentinula edodes mycelia extract (LEM) derived from the Shiitake mushroom which is an oral biological response modifier (BRM) medicine for cancer patients as an adjuvant.	47 breast cancer patients who were female, aged ≥ 20 years, were diagnosed with breast cancer, and were scheduled for anthracycline-based adjuvant chemotherapy as postoperative adjuvant therapy. The patients had a life expectancy of > 3 months.	Placebo-controlled randomized double-blind study that evaluated the effectiveness of Lentinula edodes mycelia extract (LEM).	Lentinula edodes mycelia extract (LEM), a dried powder extracted from the shiitake mushroom.	Coadministration of LEM may ameliorate anthracycline-based chemotherapy induced decreases in QOL and immune function in postoperative breast cancer patients. Oral LEM may prove to be a useful supportive care option for such postoperative breast cancer patients.
Valadares et al. 2013 (35)	Evaluate the effects of dietary supplementation of Agaricus Sylvaticus on clinical and nutritional parameters in breast cancer patients undergoing chemotherapy.	46 female patients with breast cancer, Stage II and III.	A randomized, placebo-controlled, double-blind, clinical trial (random assignment to either nutritional supplement with Agaricus Sylvaticus group or placebo group.	Agaricus Sylvaticus, also known as Blushing wood mushroom, Scaly wood mushroom, and Pinewood mushroom	Patients who were in the Agaricus Sylvaticus gooup showed improvement in gastrointestinal activities. Decreased appetite showed a 20% decrease, with no alterations in bowel functions. Vomiting and nausea also decreased in the treatment group.
COLORECTAL CANCER					
Miyake et al. 2017 (36)	Compare oral adjuvant immunochemotherapy with uracil and tegafur plus PSK (UFT/PSK) to oral adjuvant chemotherapy with uracil and tegafur plus leucovorin (UFT/LV) in stage IIB and III colorectal cancer patients to ascertain if the clinical response of UFT/PSK was “not inferior” to UFT/LV.	In this study, 351 colorectal cancer patients who also had Japanese D2/D3 lymphadenectomy. The median age was 65 years, colon/rectum stage IIB/IIIA/IIIB/IIIC.	Prospective randomized, open-label, phase III trial, with. Positive end results would indicate the patient was disease-free for 3 years. The randomized non-inferiority study compared UFT/LV to UFT/PSK. In addition to overall survival, the researchers investigated adverse events, compliance, and quality of life.	PSK (from Trametes versicolor, also known as Coriolus versicolor and Turkey Tail mushroom).	Findings could not conclude that (UFT/LV)/UFT/PSK adjuvant therapy was “non-inferior" to UFT/LV therapy. The 3-year overall disease-free survival, but secondary endpoints (adverse effects, compliance, and quality of life) showed better results with UFT/PSK than UFT/LV.

Overview

The final review considered the data provided by nine studies whereby the efficacy of medicinal mushroom extracts, e.g., polysaccharide-K (PSK), a mushroom extract also known as krestin, *Lentinus edodes* (Shiitake), *Coriolus versicolor *(Turkey Tail), and *Agaricus sylvaticu*s (Scaly Wood, Blushing Wood, or Pinewood mushroom) in treating symptoms/medication side effects, anti-tumor effects, or survival outcomes was considered. The studies on medicinal mushrooms were grouped by cancer type: breast, colorectal, and gastric. While studies exist on the therapeutic effects of mushrooms in people with lung cancer, none of the studies retrieved during the search failed to meet the study’s inclusion criteria and were therefore excluded from the final review. All nine studies reported benefits of mushroom extracts in the treatment of cancer gastric, breast, and colorectal cancers, e.g., survival‑prolonging effects, promote anti-tumor effects (gastric cancer); as an adjuvant to reduce the toxicity of chemotherapy and may thus allow for intensification of the chemotherapy dosage; improved clinical parameters of gastrointestinal functioning, such as increased appetite while undergoing chemotherapy; maintain patients’ quality of life (QoL) and immune function (breast cancer); and, as adjuvant therapy, increased QoL (colorectal cancer).

Gastric cancer

Five studies were included in the review that pertained to medicinal mushrooms and gastric cancer. Four of the five studies investigated the effect of the mushroom Turkey Tail (*Coriolus versicolor *and *Trametes versicolor*) [28-30] on people with regarding gastric cancer; the fourth study investigated Huaier (*Trametes robiniophila murr*) a sandy beige mushroom that grows on hardwood trees, and gastric cancer a [31]. Findings from these studies indicated Turkey Tail played a role in prolonging overall survival in gastric cancer patients, with few exceptions.

Turkey Tail Mushroom

Findings from the study by Hsu et al. conducted with 918 people with Stages II and III gastric cancer suggest that PSK (extracted from Turkey Tail) treatment could successfully improve the antitumor immune ability by modulating immune systems [28]. Long-term outcomes obtained in programmed death-1 ligand 1 negative (PD-L1) patients treated with PSK fared better than patients who did not receive PSK. PSK, however, had no effect on the survival of PD-L1 positive patients. Patients with negative PD-L1 who received PSK had better survival outcomes than patients who did not receive PSK, but this was not true for those with positive expression of PD-L1 (no impact on survival rate). Regarding immune response, for negative PD-L1 patients treated with PSK, percentages of natural killer and natural killer T (NKT) cells substantially increased (but not for positive PD-L1 patients). The researchers suggested that PSK produced better survival outcomes for patients with stages III and III gastric cancer, especially in the negative PD-L1 group. 

The controlled trial study by Ito et al. with 349 patients with stage II and III gastric cancer also indicated that survival outcomes were improved when PSK (extracted from Turkey Tail) was part of the therapy for patients with gastric cancer who had a curative resection and were at increased risk of recurrence due to lymph node metastasis and the absence of major histocompatibility (MHC) class I expression [29]. The findings suggest that PSK has the potential to prevent lymph node metastasis that may affect residual cancer cells that have escaped antitumor immune functions. 

Tanaka et al. also sought to evaluate the effect of PSK from Turley Tail on the overall survival of patients with gastric cancer [30]. The researchers conducted a retrospective study with 254 patients to compare the outcomes of a treatment group of PSK + Fluoropyrimidine and a control group of just Flouropurimidine therapy. Patients to whom curative surgery with postoperative adjuvant treatment with clinical stage II/III was offered were included. No significant differences between the groups were found in relapse-free or overall survival. Although, survival was improved in patients with multiple lymph node metastases following taking PSK.

Supporting these findings by Ito et al. [29] and Tanaka et al. [30], Wang et al. discovered by conducting a retrospective cohort analysis that the PSK group had significantly longer survival than the control group [31], even after adjusting for patient demographics, comorbidities, and chemotherapy regimen. The study included 10,617 patients with gastric cancers who had already had a gastrectomy and were receiving adjuvant chemotherapy. Findings from this retrospective cohort study indicated that adjuvant chemotherapy with the addition of PSK significantly reduced the risk of death in gastric cancer patients post-gastrectomy.

Huaier mushroom

The fifth and final study related to gastric cancer revealed that Huaier polysaccharides inhibit the growth of gastric cancer cells and stimulate cancer cell apoptosis [10]. The researchers retrospectively evaluated 136 stage II gastric patients. All patients reported their first diagnosis of gastric cancer after R0 resection (D2 lymph node resection) as well as having Stage IIb gastric cancer, 2) reported no other malignant neoplasia, and 3) took Tegafur Gimeracil Oteracil Potassium Capsule with/without Huaier granule as postoperative chemotherapy. Results indicated that Huaier Granule in combination with tegafur/gimeracil/oteracil improved Stage IIb gastric cancer prognosis. Patients in the study group showed greater disease-free survival (DFS) compared to the control group. Overall, findings from this study indicated that Huaier polysaccharides promote anti-tumor and gastric cell apoptosis effectively.

Breast cancer

Three studies on the therapeutic effects of medicinal mushrooms were included in the final review; two investigated shiitake mushrooms and one assessed the therapeutic effects of scaly wood mushrooms.

Shiitake Mushroom

The retrospective study by Hangai et al. analyzed data from 41 women who have pathologically proven breast cancer (stage I, IIA, or IIB) to investigate the therapeutic effects of Active hexose-correlated compound (AHCC®) on negative events in women receiving adjuvant chemotherapy for breast cancer [32]. AHCC® is a proprietary compound containing the Shiitake mushroom, containing a-glucans which are extracted from the processed mushrooms. The AHCC® group reported significantly fewer adverse neutrophil events than the control group. With taxane therapy (weekly), less usage of Granulocyte-colony stimulating factor (G-CSF) was seen in the AHCC® group than in the control group. G-CSF is a drug used to treat neutropenia, prevent infection, and prepare the blood to collect blood cells. It is used in patients who have certain cancers and neutropenia caused by various kinds of chemotherapy [33].

Nagashima and colleagues conducted a randomized double-blind study with 47 women with breast cancer who were scheduled to receive postoperative adjuvant anthracycline‑based chemotherapy. This study was performed to evaluate the effectiveness of *Lentinula edodes* mycelia extract (LEM, a dried powder extracted from shiitake mushrooms) [34]. It was found that oral LEM that was co-administered with anthracycline-based chemotherapies helped patients maintain immune function and quality of life (QoL). Overall, postoperative breast cancer patients who received the chemotherapy had decreased immune function and lowered QoL. However, when LEM was added, there was no decrease in immune function and QoL.

*Scaly Wood Mushroom* 

Valadares et al. conducted a double-blind RCT to evaluate the effects of dietary supplementation of *Agaricus sylvaticus*, also known as the Scaly Wood mushroom, in breast cancer patients undergoing chemotherapy [35]. Forty-six women with Stage II and III breast cancer were randomly assigned to receive either a nutritional supplement with *Agaricus sylvaticus* or a placebo. Patients given *Agaricus sylvaticus* experienced an improvement in clinical parameters and gastrointestinal functions. Loss of appetite decreased by 20% with no bowel function changes, vomiting, and nausea.

Colorectal cancer

Turkey Tail

The randomized, open-label, phase III trial conducted by Miyake et al. compared oral adjuvant uracil and tegafur plus leucovorin (UFT/LV) adjuvant chemotherapy to UFT/PSK adjuvant immunochemotherapy with 351 resected colorectal cancer patients [36]. The three-year disease-free survival (DFS) rate was 82.3% in patients receiving UFT/LV and 72.1% in patients receiving UFT/PSK. Findings also indicated that the non-inferiority of UFT/PSK adjuvant therapy to UFT/LV therapy was not substantiated. In addition, the three-year overall survival rate was 95.4% in those patients receiving UFT/LV and 90.7% in patients (stage IIB and III colorectal cancer patients) receiving UFT/PSK. Results from this study suggest that PSK does not have the same antitumor effects as LV. As it turned out, PSK seems like a safe immunochemotherapy agent. Regarding mitigating side effects of chemotherapy, diarrhea, and vomiting decreased significantly in the UFT/PSK group than in the UFT/LV group. Moreover, QoL increased in the UFT/PSK group more than in the UFT/LV group. Using UFT/PSK as adjuvant therapy may help patients with comorbidity and/or poor performance status. These findings combined suggest UFT/LV + PSK adjuvant chemotherapy may be considered a viable regimen for oral adjuvant chemotherapy for stage IIB and III colorectal cancer patients.

Discussion 

Nine studies addressing the therapeutic benefits of medicinal mushrooms on gastric, breast, and colorectal cancer we reported. Eight of the nine studies were conducted in Japan (n=5), Taiwan (n=2), China (n=1), and Brazil (n=1). All the studies demonstrated that the addition of certain mushrooms known as Turkey Tail, Huaier, Scaly wood, and Shiitake to anticancer chemotherapy increased survival rates, enhanced cancer cell apoptosis, and promoted anti-tumor effects within cancer patients.

One of the most well-researched medicinal mushrooms is the Turkey Tail mushroom. This mushroom contains polysaccharides, including beta-glucans, which have been shown to stimulate the immune system and have anti-tumor properties. For gastric cancer, Turkey Tail was seen to improve the antitumor immune ability by modulating immune systems, preventing lymph node metastasis, and improving survival outcomes [28-31]. Hauier was also effective in promoting antitumor effects and cell apoptosis in gastric cancer patients [10]. It is important to note, however, that patients in the studies varied regarding cancer stages and chemotherapy treatment types. For example, Polysaccharide K (PSK, the active compound in Turkey Tail) had no effect on the survival outcomes for PD-L1 positive patients but PD-L1 negative patients who received PSK had better survival outcomes than patients who did not receive PSK. While these studies indicated that taking mushrooms may help improve survival in gastric patients, most likely due to their positive effect on immune response, there may be other factors involved that could not be controlled for.

In the United States (U.S.), breast cancer is the most common cancer in women (aside from skin cancers) and its incidence is on the rise [37]. In recent years, there has been a growing interest in the role of shiitake mushrooms in breast cancer prevention and treatment. While modern medicine has made significant progress in treating breast cancer, the side effects of chemotherapy and radiation therapy can cause significant harm to the body. Therefore, complementary and alternative therapies have gained significant attention as a way to manage the side effects of conventional treatments while also improving overall health outcomes. Results from this review indicate that medicinal mushrooms, particularly Shiitake and Scaly Wood, are one such complementary therapy that has shown promise in the management of breast cancer by promoting immune function [32,34,35]. Shiitake was seen to be beneficial in reducing the side effects of breast cancer treatments. Shiitake mushrooms contain ergothioneine, a powerful antioxidant that can help to reduce oxidative stress, a common side effect of cancer treatments. It is important to note that when LEM (a dried powder extracted from shiitake mushrooms) was added to chemotherapy, there was no decrease in immune function and QoL (as is usual when undergoing chemotherapy [34]. 

Chemotherapy and radiation therapy are commonly used to treat breast cancer, but these treatments can cause side effects such as nausea, vomiting, and fatigue. Scaly Wood mushroom has shown promise for mitigating some of the effects of chemotherapy. Patients given Scaly Wood with chemotherapy experienced improvement in common side effects, with a decrease in loss of appetite, vomiting, and nausea [35]. However, none of the studies on breast cancer in this review addressed if mushrooms were effective for fatigue, a common side effect of chemotherapy in women with breast cancer [38]. It is important to note that the samples used in all three studies on breast cancer in this review were small (see limitations). Moreover, the patients were all women so it is unclear if the outcomes would differ in men with breast cancer.

Mushrooms have been studied for their potential anti-cancer properties, but it is important to note that the same mushroom may work for different cancers but in different ways. While Turkey Tail yielded positive outcomes for patients with gastric cancer, it was also seen to benefit patients diagnosed with colorectal cancer. For colorectal cancer, PSK did not change survival outcomes nor disease-free state (DFS) but adding PSK to chemotherapy mitigated some of the side effects from chemotherapy [36]. 

It was notable that no published studies on the therapeutic effects of medicinal mushrooms on lung cancer that fit the inclusion criteria for this study were found. This could be because, in the U.S., lung and bronchial cancer has the lowest survival rate of all cancers among women and men, with about half of lung cancer cases being metastatic at diagnosis [39,40]. 

Limitations

This scoping review was not without limitations. One limitation to this review process was that scoping reviews focus on providing breadth rather than depth of information on a topic, in this case, medicinal mushrooms and cancer. A meta-analysis would be helpful to ascertain actual dosages and treatment protocol recommendations specific to each cancer type and stage. A second limitation is that the included studies were limited to those published in English, so it is unknown how many relevant studies were published in other languages that were relevant to this scoping review. Third, this review was limited by the small number of available published RCTs, CTs, and retrospective cohort studies with humans regarding the use of medicinal mushrooms for major cancers (only one study on colorectal cancer was found). Fourth, all the studies on breast cancer used small sample sizes. Smaller samples may produce a result that lacks sufficient power to detect a difference between the groups, leading to a type II error. Fifth, eligible publications may have been missed as only four databases were searched and the inclusion/exclusion criteria were narrow. Finally, the inclusion criteria were limited to RCTs, CTs, and retrospective cohort studies published between January 2013- January 2023, outside of which other published reports may have been missed. 

Implications for future research

While the studies in this review suggest that medicinal mushrooms may have the potential as a complementary therapy for cancer patients, it is important to note that more research is needed to understand their effects and potential risks fully. The findings from this review suggest that medicinal mushrooms have the potential to prevent lymph node metastasis, prolong overall survival, decrease chemotherapy-induced side effects (e.g., diarrhea, vomiting), affect the immune system, and help maintain immune function and QoL in patients with certain cancers. This demonstrates the potential value of medicinal mushrooms in treating a variety of cancers. More research is needed with human subjects using RCTs with larger samples to ensure accurate outcomes. Also, rigorous research should be conducted on other deadly cancers, such as lung cancer, which grows quickly and spreads early. Lastly, more studies are needed to expand clinical trials and ascertain the most efficacious dosages and propose supplements that are genetically pure and of safe origin.

## Conclusions

Medicinal mushrooms have been found to have potential anticancer properties, with turkey tail and shiitake mushrooms being some of the most well-researched. While more research is needed, these findings suggest that medicinal mushrooms may be a promising complementary therapy for cancer patients. This scoping review highlighted the findings from recent studies with humans using mushroom extracts for use with certain types of cancers. While the current body of published research on the therapeutic effects of medicinal mushrooms in gastric, breast, and colorectal cancer in humans appears promising, it still may be in its nascent stages for practical application. However, modern medical practice is reliant on using pharmaceutical compounds whose purity can be ascertained and whose potential toxicity can be verified. The benefits of medicinal mushrooms have been recognized by traditional medicine, but more evidence-based practice research in the way of RCTs and meta-analyses may be needed before translating this traditional practice into Western medicine.

## Appendices

**Table 2:** Ovid MEDLINE(R) ALL (769 results)

1	exp Agaricales/
2	(agaric* or mushroom or mushrooms or phellinus or pleurotus or ganoderma or clitocybe or antrodia or trametes or cordyceps or xerocomus or calvatia or schizophyllum or flammulina or suillus or inonotus or inocybe or funlia or lactarius or albatrellus or russula or fomes or grifola or lentinus or lentinula or 'coriolus versicolor' or Agrocybe or Amanita or Armillaria or Coprinopsis or coprinus or Cortinarius or Cyathus or Cyclocybe or Flammulina or Grifola or Hebeloma or Hypsizygus or Laccaria or Lentinula or Macrolepiota or Marasmius or Moniliophthora or Nematoloma or Pholiota or Pleurotus or Psilocybe or Schizophyllum or Termitomyces or Tricholoma or Volvariella).mp.
3	('protein bound polysaccharide*' or psk or 'polysaccharide k').mp.
4	exp Lung Neoplasms/
5	({lung or pulmonary} adj2 {cancer* or malignan* or tum*r* or carcinoma* or neoplasm* or adenocarcino*}) .mp.
6	exp Stomach Neoplasms/
7	({stomach or gastr*} adj2 {cancer* or malignan* or tum*r* or carcinoma* or neoplasm* or adenocarcino*}).mp.
8	exp Colorectal Neoplasms/
9	({'colo rectal' or colorectal or colon or rectum or rectal} adj2 {cancer* or malignan* or tum*r* or carcinoma* or neoplasm* or adenocarcino*}).mp.
10	exp Breast Neoplasms/
11	({breast or mammary} adj2 {cancer* or malignan* or tum*r* or carcino* or neoplasm* or adenocarcino*}).mp.
12	1 or 2 or 3
13	4 or 5 or 6 or 7 or 8 or 9 or 10 or 11
14	12 and 13
15	limit 14 to yr="2012 -Current"
16	15 not (Animals/ not (Animals/ and Humans/))

**Table 3:** EMBASE Search 2-15 (1180 results)

#1	'medicinal mushroom'/exp OR 'edible mushroom'/exp OR 'agaricales'/exp OR 'mushroom'/de
#2	agaric*:ab,ti,kw OR mushroom:ab,ti,kw OR mushrooms:ab,ti,kw
#3	phellinus:ab,ti,kw OR ganoderma:ab,ti,kw OR clitocybe:ab,ti,kw OR antrodia:ab,ti,kw OR trametes:ab,ti,kw OR cordyceps:ab,ti,kw OR xerocomus:ab,ti,kw OR calvatia:ab,ti,kw OR suillus:ab,ti,kw OR inonotus:ab,ti,kw OR inocybe:ab,ti,kw OR funlia:ab,ti,kw OR lactarius:ab,ti,kw OR albatrellus:ab,ti,kw OR russula:ab,ti,kw OR fomes:ab,ti,kw OR lentinus:ab,ti,kw OR 'coriolus versicolor':ab,ti,kw OR agrocybe:ab,ti,kw OR amanita:ab,ti,kw OR armillaria:ab,ti,kw OR coprinopsis:ab,ti,kw OR cortinarius:ab,ti,kw OR cyathus:ab,ti,kw OR cyclocybe:ab,ti,kw OR flammulina:ab,ti,kw OR grifola:ab,ti,kw OR hebeloma:ab,ti,kw OR hypsizygus:ab,ti,kw OR laccaria:ab,ti,kw OR lentinula:ab,ti,kw OR macrolepiota:ab,ti,kw OR marasmius:ab,ti,kw OR moniliophthora:ab,ti,kw OR nematoloma:ab,ti,kw OR pholiota:ab,ti,kw OR pleurotus:ab,ti,kw OR psilocybe:ab,ti,kw OR schizophyllum:ab,ti,kw OR termitomyces:ab,ti,kw OR tricholoma:ab,ti,kw OR volvariella:ab,ti,kw
#4	'protein bound polysaccharide*':ab,ti,kw OR psk:ab,ti,kw OR 'polysaccharide k':ab,ti,kw
#5	#1 OR #2 OR #3 OR #4
#6	'lung cancer'/exp
#7	(lung OR pulmonary) NEAR/2 (cancer* OR malignan* OR tum*r* OR carcinoma* OR neoplasm* OR adenocarcino*)
#8	'stomach cancer'/exp
#9	(stomach OR gastr*) NEAR/2 (cancer* OR malignan* OR tum*r* OR carcinoma* OR neoplasm* OR adenocarcino*)
#10	'colorectal cancer'/exp
#11	('colo rectal' OR colorectal OR colon OR rectum OR rectal) NEAR/2 (cancer* OR malignan* OR tum*r* OR carcinoma* OR neoplasm* OR adenocarcino*)
#12	'breast cancer'/exp
#13	(breast OR mammary) NEAR/2 (cancer* OR malignan* OR tum*r* OR carcino* OR neoplasm* OR adenocarcino*)
#14	#6 OR #7 OR #8 OR #9 OR #10 OR #11 OR #12 OR #13
#15	#5 AND #14
#16	#15 NOT ('animals'/exp NOT 'humans'/exp) AND 2012-2023/py

**Table 4:** CINAHL Search 2-15 (201 results)

S1	(MH "Mushroom") OR (MH "Mushroom, Edible")
S2	TI ( (agaric* or mushroom or mushrooms or phellinus or pleurotus or ganoderma or clitocybe or antrodia or trametes or cordyceps or xerocomus or calvatia or schizophyllum or flammulina or suillus or inonotus or inocybe or funlia or lactarius or albatrellus or russula or fomes or grifola or lentinus or lentinula or 'coriolus versicolor' or Agrocybe or Amanita or Armillaria or Coprinopsis or coprinus or Cortinarius or Cyathus or Cyclocybe or Flammulina or Grifola or Hebeloma or Hypsizygus or Laccaria or Lentinula or Macrolepiota or Marasmius or Moniliophthora or Nematoloma or Pholiota or Pleurotus or Psilocybe or Schizophyllum or Termitomyces or Tricholoma or Volvariella) ) OR AB ( (agaric* or mushroom or mushrooms or phellinus or pleurotus or ganoderma or clitocybe or antrodia or trametes or cordyceps or xerocomus or calvatia or schizophyllum or flammulina or suillus or inonotus or inocybe or funlia or lactarius or albatrellus or russula or fomes or grifola or lentinus or lentinula or 'coriolus versicolor' or Agrocybe or Amanita or Armillaria or Coprinopsis or coprinus or Cortinarius or Cyathus or Cyclocybe or Flammulina or Grifola or Hebeloma or Hypsizygus or Laccaria or Lentinula or Macrolepiota or Marasmius or Moniliophthora or Nematoloma or Pholiota or Pleurotus or Psilocybe or Schizophyllum or Termitomyces or Tricholoma or Volvariella) )
S3	TI ('protein bound polysaccharide*' or psk or 'polysaccharide k') OR AB ( ('protein bound polysaccharide*' or psk or 'polysaccharide k')
S4	S1 OR S2 OR S3
S5	(MH "Lung Neoplasms+")
S6	TI ({lung or pulmonary} N2 {cancer* or malignan* or tum*r* or carcinoma* or neoplasm* or adenocarcino*}) OR AB ({lung or pulmonary} N2 {cancer* or malignan* or tum*r* or carcinoma* or neoplasm* or adenocarcino*})
S7	(MH "Stomach Neoplasms")
S8	TI ({stomach or gastr*} N2 {cancer* or malignan* or tum*r* or carcinoma* or neoplasm* or adenocarcino*} OR AB {stomach or gastr*} N2 {cancer* or malignan* or tum*r* or carcinoma* or neoplasm* or adenocarcino*})
S9	(MH "Colorectal Neoplasms+")
S10	TI ({'colo rectal' or colorectal or colon or rectum or rectal} N2 {cancer* or malignan* or tum*r* or carcinoma* or neoplasm* or adenocarcino*} OR AB {'colo rectal' or colorectal or colon or rectum or rectal} N2 {cancer* or malignan* or tum*r* or carcinoma* or neoplasm* or adenocarcino*})
S11	(MH "Breast Neoplasms+")
S12	TI ({breast or mammary} N2 {cancer* or malignan* or tum*r* or carcino* or neoplasm* or adenocarcino*} OR AB {breast or mammary} N2 {cancer* or malignan* or tum*r* or carcino* or neoplasm* or adenocarcino*})
S13	S5 OR S6 OR S7 OR S8 OR S9 OR S10 OR S11 OR S12
S14	S4 AND S13
S15	S14 Limiters – Published Date: 2012-2023

**Table 5:** Alt HealthWatch 2-15 (52 results)

S1	MH "mushrooms" OR MH "agaricus"
S2	TI ({agaric* or mushroom or mushrooms or phellinus or pleurotus or ganoderma or clitocybe or antrodia or trametes or cordyceps or xerocomus or calvatia or schizophyllum or flammulina or suillus or inonotus or inocybe or funlia or lactarius or albatrellus or russula or fomes or grifola or lentinus or lentinula or 'coriolus versicolor' or Agrocybe or Amanita or Armillaria or Coprinopsis or coprinus or Cortinarius or Cyathus or Cyclocybe or Flammulina or Grifola or Hebeloma or Hypsizygus or Laccaria or Lentinula or Macrolepiota or Marasmius or Moniliophthora or Nematoloma or Pholiota or Pleurotus or Psilocybe or Schizophyllum or Termitomyces or Tricholoma or Volvariella} OR AB ( (agaric* or mushroom or mushrooms or phellinus or pleurotus or ganoderma or clitocybe or antrodia or trametes or cordyceps or xerocomus or calvatia or schizophyllum or flammulina or suillus or inonotus or inocybe or funlia or lactarius or albatrellus or russula or fomes or grifola or lentinus or lentinula or 'coriolus versicolor' or Agrocybe or Amanita or Armillaria or Coprinopsis or coprinus or Cortinarius or Cyathus or Cyclocybe or Flammulina or Grifola or Hebeloma or Hypsizygus or Laccaria or Lentinula or Macrolepiota or Marasmius or Moniliophthora or Nematoloma or Pholiota or Pleurotus or Psilocybe or Schizophyllum or Termitomyces or Tricholoma or Volvariella) )
S3	TI ({'protein bound polysaccharide*' or psk or 'polysaccharide k'} OR AB {'protein bound polysaccharide*' or psk or 'polysaccharide k'} )
S4	S1 OR S2 OR S3
S5	MH "lung cancer"
S6	TI ({lung or pulmonary} N2 {cancer* or malignan* or tum*r* or carcinoma* or neoplasm* or adenocarcino*} OR AB {lung or pulmonary} N2 {cancer* or malignan* or tum*r* or carcinoma* or neoplasm* or adenocarcino*}
S7	MH "stomach cancer"
S8	TI ({stomach or gastr*} N2 {cancer* or malignan* or tum*r* or carcinoma* or neoplasm* or adenocarcino*} OR AB {stomach or gastr*} N2 {cancer* or malignan* or tum*r* or carcinoma* or neoplasm* or adenocarcino*})
S9	MH "colorectal cancer"
S10	TI ({'colo rectal' or colorectal or colon or rectum or rectal} N2 {cancer* or malignan* or tum*r* or carcinoma* or neoplasm* or adenocarcino*} OR AB {'colo rectal' or colorectal or colon or rectum or rectal} N2 {cancer* or malignan* or tum*r* or carcinoma* or neoplasm* or adenocarcino*})
S11	MH "breast cancer"
S12	TI ({breast or mammary} N2 {cancer* or malignan* or tum*r* or carcino* or neoplasm* or adenocarcino*} OR AB {breast or mammary} N2 {cancer* or malignan* or tum*r* or carcino* or neoplasm* or adenocarcino*})
S13	S5 OR S6 OR S7 OR S8 OR S9 OR S10 OR S11 OR S12
S14	S4 AND S13
S15	S14 Limiters – Published Date: 2012-2023
