# Correction: Therapeutic Effects of Medicinal Mushrooms on Gastric, Breast, and Colorectal Cancer: A Scoping Review

**DOI:** 10.7759/cureus.c309

**Published:** 2025-09-23

**Authors:** Amit Dan, Robyn Swain, Seigna Belonce, Robin J Jacobs

**Affiliations:** 1 Department of Nutrition, Nova Southeastern University Dr. Kiran C. Patel College of Osteopathic Medicine, Fort Lauderdale, USA; 2 Departments of Research/Health Informatics/Nutrition, Nova Southeastern University Dr. Kiran C. Patel College of Osteopathic Medicine, Fort Lauderdale, USA

This article has been corrected to fix the following typo in the PRISMA diagram: Records identified from OVID Medline corrected from 796 to 769.

